# Deaf-Mutes and Consanguineous Marriages

**Published:** 1887-02-19

**Authors:** 


### DEAF-MUTES AND CONSANGUINEOUS MARRIAGES.

THE Foreign Office, in answer to a circular of Lord Salisbury's, has lately published a series of interesting papers on the state of deaf-mutes in other countries. The returns from the United States contain some curious information regarding heredity as a cause of "deaf-mutism," the *St. James's Gazette* states. Though the great majority of children of deaf-mute parents are not deaf, and many deaf-mutes have no deaf offspring, the proportion of deaf children is much greater with deaf parents than with hearing parents. Nevertheless, it is noted that the proportion is higher where one only of the parents is deaf than where both are deaf, unless the deafness is congenital. Thus, of 162 deaf-mutes married to hearing persons, 55 having deaf-mute relatives had 15 deaf children; but of the rest, 107, who had not deaf relatives, had only one deaf child. Consanguineous marriages, it is said, only tend to intensify tendencies, whether for good or for ill; and the probabilities of hereditary deaf-mutism do not necessarily vary with the degree of consanguinity. The figures from four schools of pupils admitted in 1877 are thus given :—

| | | | | |
|---|---|---|---|---|
| Parents: First cousins...... | 45 families | yield | 63 | deaf-mutes. |
| „ Second cousins .. | 10 | „ | 19 | „ |
| „ Third cousins .... | 7 | „ | 11 | „ |
| „ Fourth cousins .. | 2 | „ | 3 | „ |

And one uncle and niece produced one deaf-mute.

